# Increased Mammogram-Induced DNA Damage in Mammary Epithelial Cells Aged *In Vitro*

**DOI:** 10.1371/journal.pone.0063052

**Published:** 2013-05-07

**Authors:** Laia Hernández, Mariona Terradas, Marta Martín, Purificación Feijoo, David Soler, Laura Tusell, Anna Genescà

**Affiliations:** Department of Cell Biology, Physiology, and Immunology, Universitat Autònoma de Barcelona, Bellaterra, Spain; University of Nebraska Medical Center, United States of America

## Abstract

Concerned about the risks of mammography screening in the adult population, we analyzed the ability of human mammary epithelial cells to cope with mammogram-induced DNA damage. Our study shows that an X-ray dose of 20 mGy, which is the standard dose received by the breast surface per two-view mammogram X-ray exploration, induces increased frequencies of DNA double-strand breaks to *in vitro* aged–but not to young–human mammary epithelial cells. We provide evidence that aged epithelial breast cells are more radiosensitive than younger ones. Our studies point to an inefficient damage response of aged cells to low-dose radiation, this being due to both delayed and incomplete mobilization of repair proteins to DNA strand breaks. This inefficient damage response is translated into an important delay in double-strand break disappearance and consequent accumulation of unrepaired DNA breaks. The result of this is a significant increase in micronuclei frequency in the *in vitro* aged mammary epithelial cells exposed to doses equivalent to a single mammogram X-ray exploration. Since our experiments were carried out in primary epithelial cell cultures in which cells age at the same time as they undergo replication-dependent telomere shortening, we needed to determine the contribution of these two factors to their phenotype. In this paper, we report that the exogenous expression of human telomerase retrotranscriptase in late population doubling epithelial cells does not rescue its delayed repair phenotype. Therefore, retarded DNA break repair is a direct consequence of cellular aging itself, rather than a consequence of the presence of dysfunctional telomeres. Our findings of long-lasting double strand breaks and incomplete DNA break repair in the *in vitro* aged epithelial cells are in line with the increased carcinogenic risks of radiation exposures at older ages revealed by epidemiologic studies.

## Introduction

Breast cancer mortality is declining in many western countries. Both the improved effectiveness of treatment and mammography-screening programs, which involve women aged 50–70 years in most western countries, have contributed to decreasing this rate. However, like almost all medical procedures, regular screening mammography in woman brings benefits as well as risks. In all European countries, the breast cancer rate has increased in parallel with the dissemination of mammographies, without significantly reducing the incidence of aggressively growing tumors [1], [2]. Therefore, one concern surrounding mammography screening is the possibility that the radiation received from the regular screening of mammograms may ultimately induce cancer.

Epidemiological studies provide evidence of increased breast cancer risks in populations exposed to low or moderate radiation doses for medical reasons. Elevated breast cancer risks have been reported in women who received repeated fluoroscopic examinations for tuberculosis [3] or for a population that had undergone frequent X-ray examinations for spinal curvature [4]. Furthermore, elevated breast cancer risk has been reported amongst women who had multiple chest X-rays or mammograms 5 years or more before diagnosis [5]. However, due to the limited sensitivity of epidemiological studies, current mammogram-risk figures derive from epidemiological datasets with populations exposed to higher radiation doses. This extrapolation from high-to-low radiation doses is based on the unproven assumption that the extent of damage to a cell genome is proportionate to the radiation dose received, even when the dose is very low. However, some authors claim that, after low-dose radiation exposures such as mammogram X-ray doses, cells cannot efficiently respond to DNA lesions (reviewed in [6]). The concept of threshold for repair triggering gained support from the observation that fibroblasts fail to repair DSBs when they contain less than one DSB for each 20 cells [7] and also that radiation doses inducing less than ∼20 DSBs (<0.4 Gy) fail to initiate the G2/M checkpoint [8].

Adding yet more complexity to this scenario, epidemiological studies have shown that there are important age-related differences in sensitivity to ionizing radiation in the human population, children and older people being the most sensitive. In Hiroshima and Nagasaki bomb survivor cohorts, radiation-induced cancer risks decreases with increasing age at exposure only until exposure ages of 30–40 years; at older ages, this risk increases for many individual cancer sites, as well as for all solid cancers combined [9]. Similar epidemiological evidence has been obtained for adult exposures to low-dose radiation. Studies of nuclear-plant workers have provided evidence for a positive association between age at exposure and carcinogenic risk of radiation as they reveal a stronger dose-effect relationship for doses received at older ages [10]–[13]. All these observations raise the question of whether low-dose mammogram X-ray exposures could induce increased DNA damage in aged breast cells. We considered the possibility that the accumulation of dysfunctional telomeres in aged cells or a progressive impairment of responses triggered by cells when faced to DNA lesions (so called DNA damage response, DDR) could contribute to increasing the risk of radiation exposures in the elderly. Telomere erosion enhances high-dose radiation sensitivity because uncapped chromosomes can interfere with the correct repair of radiation-induced double strand breaks (DSBs) [14]. Additionally, it should be taken into account that impairment of cell cycle control and DNA repair mechanisms have been reported in aged fibroblasts and lymphocytes [15]–[17]. In order to avoid the uncertainty that can arise when carcinogenic risks are extrapolated from one cell type to another, it is important to investigate the molecular basis underlying age-dependent variations in the carcinogenic risks of radiation directly in epithelial cells, from which most breast cancers originate.

To gain insight into the carcinogenic risks of mammogram screening and the modulation of such risks through cell aging, we used epithelial cells derived from normal human mammary specimens that were then irradiated under a mammogram device. We examined the formation of γH2AX foci to estimate DSB induction and disappearance over time after radiation exposure in proliferating (non-senescent) *in vitro* aged versus young mammary epithelial cells. In order to approach mechanistic clues underlying the observed differences, we examined DNA break disappearance after restoring telomerase activity, as well as recruitment of 53BP1*–*an important cell cycle and repair factor*–*to sites of lesions. Of relevance, our analyses show an increased induction of DSBs after X-ray doses equivalent to those received during mammogram explorations and a diminished capacity of *in vitro* aged epithelial breast cells to cope with DNA damage, a scenario that is not reversed by telomerase reactivation.

## Results

### A Single Mammogram X-ray Exploration Induces Increased Frequencies of DSBs to in vitro Aged–but not to Young–human Mammary Epithelial Cells

Women aged 50–70 years included in most national breast-screening programs receive a two-view exposure (oblique and craniocaudal) every 1, 2 or 3 years. The mean glandular dose in the general population (per two-view screening examination) is 4.5 mGy and the standard entrance skin dose is 20 mGy. Epidemiological studies are not sensitive enough to detect risks below 50 mGy [18] making it necessary to carry out an experimental risk assessment to overcome this limit. Consequently, we initially reflected on whether mammogram X-ray explorations produced a detectable increase of DSBs in the cells derived from mammary gland. We also considered whether there were differences between early and late population doubling (PD) cell samples. Our analysis focused on this type of DNA lesion because it plays a crucial role in human carcinogenesis. In order to minimize the impact of senescent cells, which have already triggered a mechanism that stops incipient cancer cells from proliferating, we used early and late PD HMEC samples containing fewer than 10% senescent cells in all experiments *–*as demonstrated by a β-galactosidase assay.

**Figure 1:**
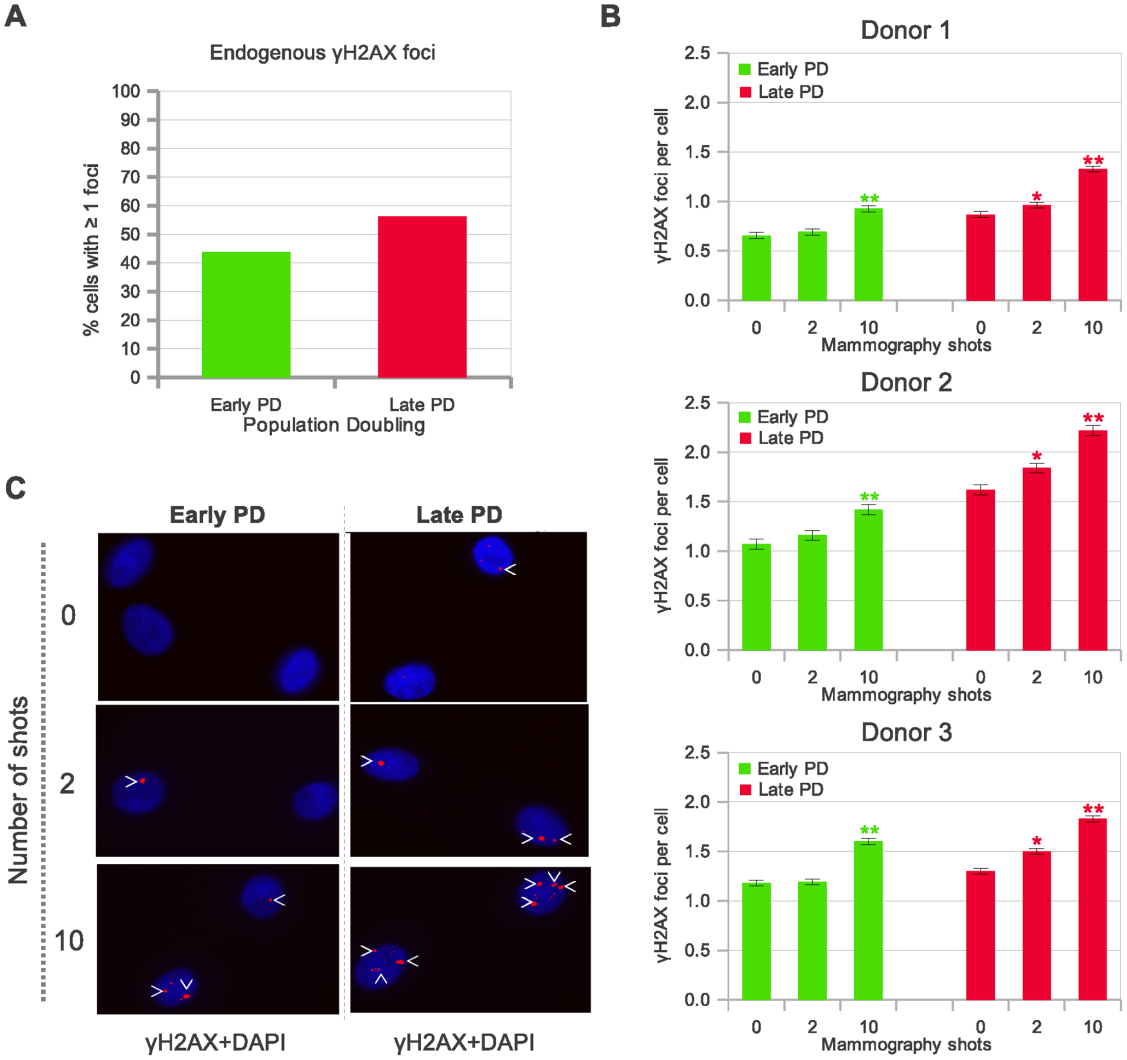
Detection of phosphorylated histone (γH2AX) foci in human mammary epithelial cells (HMECs) to estimate DNA damage. **A.** Endogenous γH2AX foci: the proportion of cells containing ≥1 foci in late PD cells is significantly higher than in early PD cells (p<0.0001, *Chi*-squared test). The foci were counted in 1000 cells per sample. **B.** Mean incidence of γH2AX foci per cell 2 h after mammogram X-ray exposures is greater in the late PD HMECs in each of the three donors analyzed. The foci were counted in 2000 cells (donor 1) and 1000 cells (donor 2 and 3) per group. Error bars signify standard error. Asterisk denotes statistically significant difference in a group of irradiated HMECs compared to the shamirradiated controls of each cell subpopulation (Mann Whitney test). Simple asterisk (*) refers to statistically significant difference p<0.01 and double asterisk (**) refers to highly significant difference p<0.0001. **C.** Representative images of γH2AX foci in HMECs from early and late PD exposed to 0, 2 and 10 automatic X-ray shots under a mammogram device (2 hours post-irradiation). Arrowheads point to the γH2AX foci stained in red. *In vitro* aged populations exhibited a higher number of γH2AX foci compared to the young counterparts.

First, we used γH2AX (protein immunofluorescence) detection to identify and quantify basal levels of DSBs in epithelial mammary cells derived from three different healthy donors. The nucleosomal histone H2AX is phosphorylated on its Ser139 in large segments of DSB-flanking chromatin, which are visible by epifluorescent microscopy as nuclear foci [19], [20]. We observed that the proportion of cells containing endogenous γH2AX foci was higher in the *in vitro* aged cell subpopulations (Figure 1A shows results obtained in HMECs derived from donor 1; *chi*-squared test, p<0.0001). The higher levels of endogenous damage in late PD cells in comparison to their early counterparts may be revealing age-associated accumulation of irreparable DSBs [17], [21] and/or critical telomere erosion [22].

In order to assess DSB induction by mammography screening, early and late PD HMEC samples from three different donors were irradiated with 2 and 10 automatic-shot X-rays under a mammogram device (10 mGy per shot; doses equivalent to 1 and 5 two-view screens, respectively). The time interval between shots was under 30 seconds. To avoid observer bias, scoring of γH2AX foci was performed blindly on coded slides. The analyses were carried out 120 min after radiation exposure. We selected this timing because it was reported as optimal for obtaining a maximum γH2AX foci scoring in most cell types [23]. If the early and late PD HMECs had had the same radiation sensitivity, we should have found similar increases in the frequencies of DSBs after mammogram X-ray exposure. Instead, we found that there was a more pronounced increase between shamirradiated and 10 shot in late PD cells than in early PD cells in each of the three donor samples analysed (Figure 1B and 1C). Most importantly, only 2 shots were sufficient to show a statistically significant increased amount of damage in the *in vitro* aged cells, but not in the young counterparts (Mann Whitney test, p<0.05 each sample).

### Mammogram-induced DSBs in Late PD Cells Remain Unrepaired for Longer Times, thus Promoting Illegitimate End Joining

**Figure 2:**
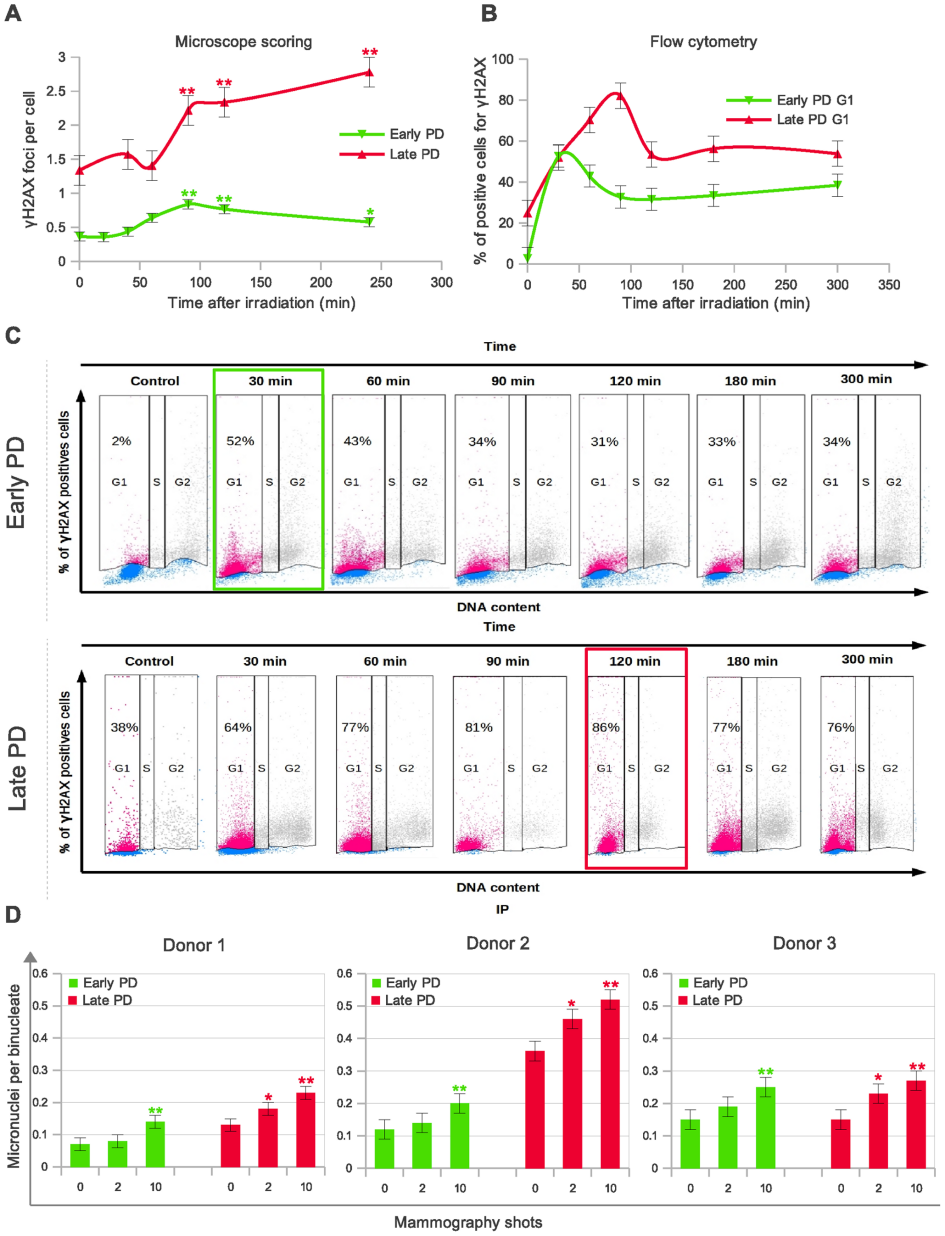
DNA double strand breaks (DSBs) measured by detection of γH2AX induction in late PD HMECs by mammogram X-rays last longer than in early PD HMECs. **A.** Time-dependence of γH2AX foci formation and disappearance measured by direct microscopic visualization. The foci were counted in 1000 cells per each time and cell subpopulation group. Error bars signify standard error. Simple asterisk (*) refers to statistically significant difference p<0.05 and double asterisk (**) refers to highly significant difference p<0.0001. Mann Whitney test was performed in all samples. **B.** Time-dependent flow cytometric bivariant detection of propidium iodide (PI)-and γH2AX. Histogram showing the kinetics of γH2AX appearance and disappearance measured by flow cytometry. For early PD HMECs (green), the maximum percentage of γH2AX positive G1-stage cells was reached at 30 minutes after irradiation, whereas *in vitro* aged HMECs (red) do not reach maximum until 90 min time-point. A minimum of 10,000 cells and two replicas were analyzed by bivariate flow cytometry for each time-point and cell subpopulation. **C.** Representative dot-plots illustrate that γH2AX labeling lasts longer in the *in vitro* aged epithelial cells (red dots indicate positive and blue negative γH2AX labeled cells). Cell cycle is displayed using contour plots and γH2AX labeling using pseudo-color plots. Gating set at 3% increment over unspecific background fluorescence for both control and irradiated HMECs. Percentages of positive γH2AX cells at the G1 cell cycle stage are indicated in each dot-plot. For cytometric analysis, early and late PD HMECs were irradiated with 1Gy γ-rays. **D.** Mean incidence of micronuclei per cell in early and late PD HMECs derived from three different donors and exposed to 0, 2 and 10 automatic X-ray shots under a mammogram device (24 h after irradiation). The micronuclei were counted in 2000 (donor 1) and 500 (donor 2 and 3) binucleates per group. Error bars signify standard error. Asterisk denotes statistically significant difference in a group of irradiated HMECs compared to the shamirradiated controls of each cell subpopulation (Mann Whitney test). Simple asterisk (*) refers to statistically significant difference p<0.05 and double asterisk (**) refers to highly significant difference p<0.0001.

In order to broaden our understanding of the age cell-dependence of low-dose radiation sensitivity, we carried out a time-course experiment to score the number of γH2AX foci at 6 different times post-IR in early and late PD HMECs. It is accepted that rapid loss of γH2AX is contingent upon functional and efficient DSB repair [20]. The results of γH2AX foci dynamics after 10 automatic-shot X-rays under a mammogram device are shown in Figure 2A. It can be deduced from these experiments that the disappearance of γH2AX foci is strongly dependent on the cell’s PD. Early PD HMECs have a maximum peak of γH2AX foci 90 min post-irradiation, with the number of foci decreasing significantly thereafter. In contrast, the mean number of γH2AX foci in late PD increases throughout the whole experiment (4 hours), suggesting that DSBs remain unrepaired for a more prolonged time in the *in vitro* aged mammary epithelial cells than in their young counterparts.

Direct microscopic visualization of γH2AX is probably the most specific and sensitive technique for spotting DSBs in cells and for monitoring their repair. However, as it is highly time-consuming, the number of cells analyzed for each time-point is limited. Therefore, we also analyzed the kinetics of damage removal by flow cytometry. To increase the sensitivity of this assay, we irradiated the cells with 1 Gy γ-rays. We combined a fluorescently labeled antibody against γH2AX and DNA staining with propidium iodide (PI), since it is known that the frequency of γH2AX positive cells varies according to the phase of the cell cycle [23]. Through this assay, we were able to measure changes in the level of the phospho-histone in relation to the cell-cycle position in individual cells within each subpopulation. Flow cytometric analysis of γH2AX labeling in G1-phase cells was more sensitive than in other cell cycle phases because background staining is lower. Analysis of the kinetics of γH2AX appearance and disappearance in G1 HMECs by flow cytometry confirmed the results obtained through microscope analysis. Figure 2B shows the average percentage of γH2AX positive G1 cells, which reached a maximum at 30 minutes after irradiation in early PD HMECs, whereas *in vitro* aged HMECs do not reach maximum until 90 min post-irradiation. After these time-points a decrease in γH2AX labeling was observed in all samples, but was far more moderated in the *in vitro* aged cell samples. Figure 2C, shows a representative example of the dot-plots obtained for early and late PD HMECs in one of the experiments. DSBs that remain open for longer can potentially promote cytogenetic damage. In agreement with this possibility, a significant increase in micronucleus frequency in binucleated HMECs from three different donors was observed in late PD –but not in their early counterparts– after only two shot mammogram X-rays (Figure 2D, p<0.05 for each donor sample; test Mann-Whitney). This higher frequency of micronuclei in the late PD cell samples is probably a consequence of illegitimate rejoining promoted by long lasting DSBs that accumulate in cells. A similar situation has been reported in repair deficient cell models [24]. In summary, DNA breaks remain unrepaired for longer in the *in vitro* aged cell samples, resulting in increased cytogenetic damage after exposure doses equivalent to a single mammogram exploration.

### Delayed Disappearance of DSBs in Late PD Samples is Directly Linked to Cell Aging rather than to the Dysfunctional Telomeres Present in Aged Cells

**Figure 3:**
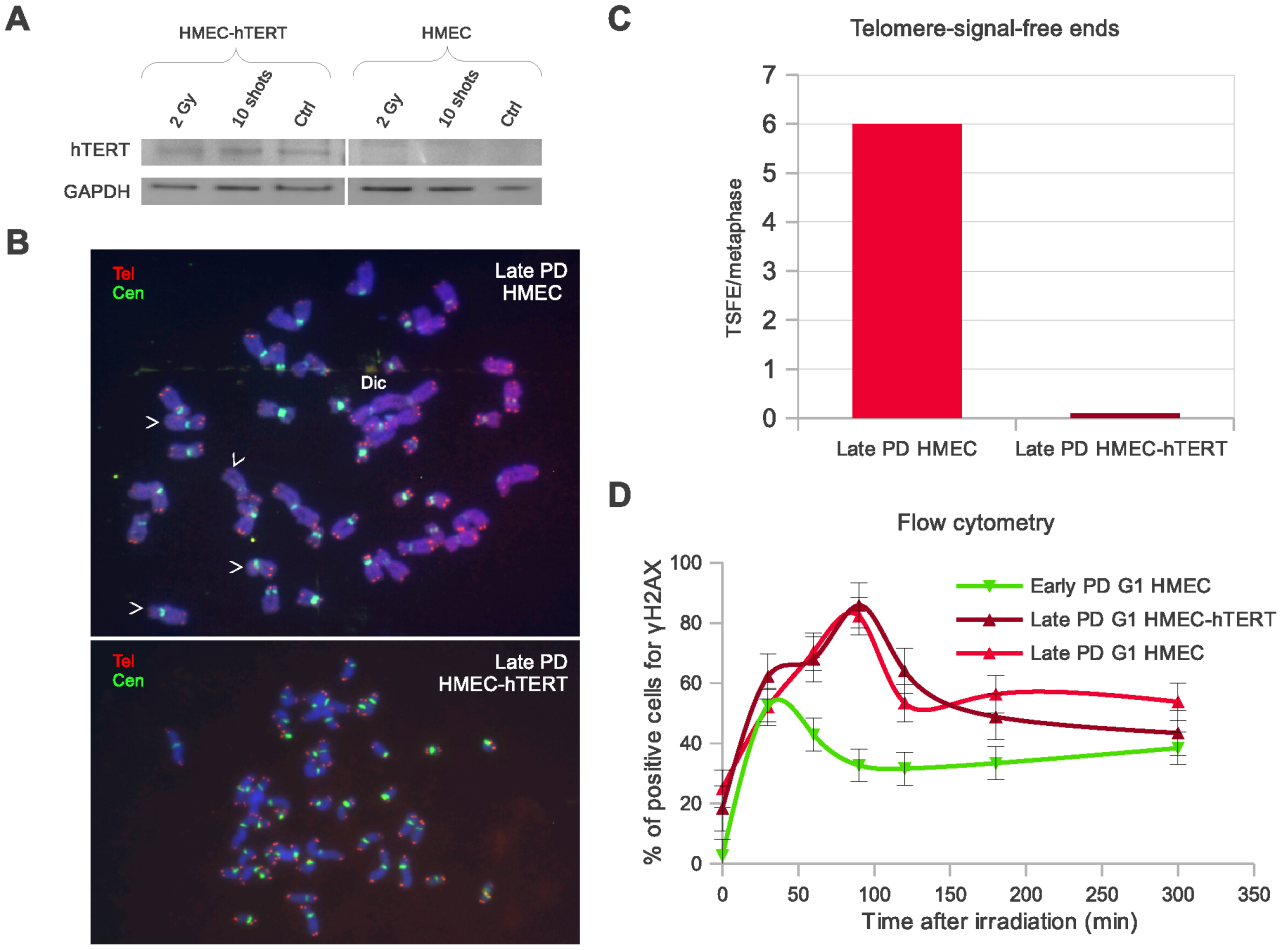
Restoration of telomerase activity in the late PD HMEC samples does not rescue their delayed DSB disappearance phenotype. Results obtained from donor 1 samples. **A.** Western blot showing that transduction of late PD HMECs with *hTERT* gene restituted telomerase protein subunit expression. **B.** Metaphase plates of a late PD non-transduced (top) and *hTERT*-transduced (bottom) HMECs. Telomeric (red) and centromeric (green) sequences detected with fluorescent *in situ* hybridization procedures. Telomerase restoration resulted in a reduction of telomere-signal-free ends (white arrow heads) and end-to-end fusions (Dic) as a consequence of telomere elongation. **C.** Bar diagram showing average frequencies of telomere-signal-free ends in non-transduced and *hTERT*-transduced HMECs. **D.** Histogram showing the kinetics of γH2AX appearance and disappearance measured by flow cytometric analysis. *hTERT*-transduced late PD HMECs (brown) reach its maximum percentage of γH2AX positive cells 90 min post-irradiation, which is delayed as compared to the early PD HMECs (green), but is the same time point as non-transduced late PD HMECs (red). A minimum of 10,000 cells and two replicas were analyzed by bivariate flow cytometry for each time-point and cell subpopulation.

The presence of dysfunctional telomeres in aged proliferative cells is associated with increased radiosensitivity as they interfere with the correct joining of DNA strand breaks [14]. We therefore speculated on whether the observed delay in DSB disappearance in the late PD cell samples was caused by the presence of dysfunctional telomeres or, instead, whether it was a direct consequence of cell aging. For this purpose, we investigated whether DSB repair kinetics–as measured by γH2AX and PI bivariant flow cytometry– was influenced by the restoration of telomerase through transduction of *hTERT* gene in late PD HMECs derived from donor 1 (Figure 3A). To ensure we had transduced the cells properly, instead of measuring the average telomere lengthening, we scored the frequency of chromosome ends without visible telomere signals (probably uncapped ends). We observed a significant reduction in the frequency of chromosome ends with no visible telomeric signal after transduction of cells with *hTERT* (Figure 3B and 3C). Regardless of having been transduced (or not) with *hTERT*, all late PD HMECs reached the maximum percentage of γH2AX positive G1-stage cells with delay, at 90 minutes after irradiation (Figure 3D). Taken as a whole, this would suggest that rather than a consequence of the presence of dysfunctional telomeres, the delayed disappearance of DSBs in the *in vitro* aged samples is directly associated with chronological cell aging.

### Deficient Response of Aged Mammary Epithelial Cells to Mammogram-induced DNA Damage

**Figure 4:**
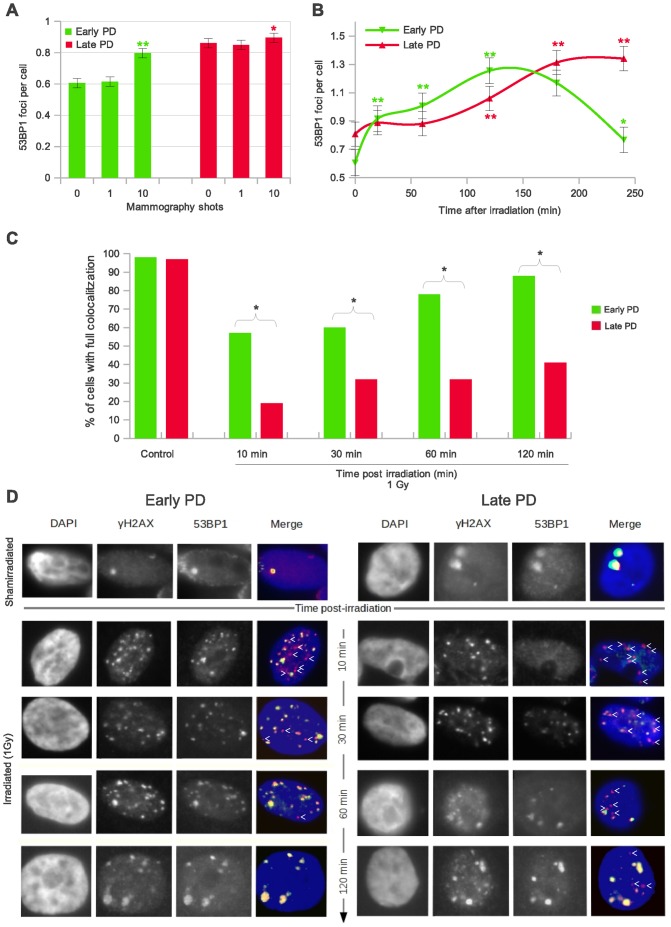
Mammogram-induced DNA damage response in early and late PD HMECs. **A.** Mean incidence of 53BP1 foci per cell 120 min after mammogram X-ray exposures: weakened low-dose radiation response of late PD HMECs. The foci were counted in 2000 cells per group (donor 1). Error bars signify standard error. Asterisk denotes statistically significant difference in a group of irradiated HMECs compared to the shamirradiated controls of each subpopulation. **B.** Diagram showing the kinetics of 53BP1 foci formation. Late PD HMECs show a 100-minute delay in their peak of 53BP1 foci per cell as compared to early PD HMECs. The 53BP1 foci for kinetics analyses were counted in 1000 cells per time-point and cell subpopulation (donor 1). Cells were exposed to 10 automatic X-ray shots under a mammogram device. **A & B.** Error bars signify standard error. Simple asterisk (*) refers to statistically significant difference p<0.05 and double asterisk (**) refers to highly significant difference p<0.0001. Mann Whitney test was performed in all samples. **C.** Histogram showing the fraction of cells with full colocalitzation of γH2AX and 53BP1 for both early and late PD in time (donor 1). Late PD samples do not reach full colocalitzation even after 2 h post-irradiation, revealing a slower mobilization of repair proteins to the damaged site than early PD HMECs. **D.** Representative images of early and late PD HMECs immunostained for γH2AX and 53BP1 at various times post-IR. Cells were irradiated with 1Gy of γ-rays. Red (γH2AX) and green (53BP1) fluorochromes appear yellow where they coincide in the merged images. Post-IR mobilization of 53BP1 to the γH2AX nuclear foci follows different kinetics in early and late PD HMECs.

To approach mechanistic clues underlying the observed radiation sensitivity of late PD epithelial mammary cells, we investigated their capacity to trigger an effective response when exposed to low doses of X-ray. The reduced amount of DSBs induced by mammograms may pose a serious threat to genome integrity if they are not sufficient to trigger DDR. We were therefore led to enquire whether mammary epithelial cells do indeed respond to the low number of DSBs induced by mammogram X-ray exposures, and whether early and late PD cells do this with the same efficacy. To answer these questions, we blindly analyzed the formation of 53BP1 foci, a protein that participates in the activation of factors involved in cell-cycle control and DNA repair if recruited at break sites [25]. In the early PD HMECs irradiated under the mammogram device (0, 1 and 10 shots), enumeration of 53BP1 discrete foci revealed a significantly increase with respect to the basal levels after 10 automatic shots (Mann Whitney<0.001; Figure 4A). This increase is in line with the observed increase of γH2AX foci (Figure 1B) thus providing evidence of an effective response despite the small number of DSBs induced. In contrast to the early passage cell samples, the previously observed increase in the number of γH2AX foci after two X-ray shots (Figure 1B) did not entail a significant increase of 53BP1 foci in late passage HMECs (Mann Whitney p>0.05; Figure 4A).

The results obtained by 53BP1 foci scoring may indicate that late PD cells require a higher number of lesions to trigger DDR or, alternatively, that the response exists but is delayed. To address this question, we needed to investigate the kinetics of DSB response. We irradiated early and late passage HMECs from donor 1 with 10-shot mammogram exposures, and quantified 53BP1 foci at five different time-points after irradiation and in the shamirradiated control (Figure 4B). The results of our time-course experiment indicate that early PD cells are able to recruit 53BP1 at the damage site faster than their late PD counterparts. The first time-point showing an increase in 53BP1 recruitment to the sites of damage was 20 min in early passage cells and 120 min after irradiation in late passage cells (Figure 4B; Mann Whitney test, p<0.001). The maximum frequency of 53BP1 foci in the late PD HMECs was 100 min delayed, which points to inefficient DDR protein recruitment in the *in vitro* aged epithelial cells.

In order to fully determine that DDR efficiency decreases with cell aging, a time-course experiment for measuring co-localization of γH2AX and 53BP1 foci after 1Gy X-rays was performed. The results of the time-course experiment are summarized in Figure 4C and are illustrated in Figure 4D. Complete colocalization between γH2AX and 53BP1 endogenous foci was observed in almost 100% of cells in both shamirradiated subpopulations. In contrast, statistically significant differences between early and late PD samples were observed in every time-point after irradiation (p<0.0001 for each time-point analyzed; *Chi*-square test). Barely 10 min after irradiation, 50% of early PD cells showed full colocalization of γH2AX and 53BP1 signals whilst less than 20% of late PD HMECs accomplished this. The percentage of cells having full colocalization increased over time in both cell samples; importantly, however, nearly 100% of the early PD samples achieved full colocalization 2 h after damage infliction, whereas this remained under 50% in late PD samples. Recruitment of 53BP1 into the damage sites is therefore not only delayed but is also incomplete in late PD samples. The inability of the *in vitro* aged HMECs to recruit repair factors in an efficient and complete way may–jointly with the presence of dysfunctional telomeres that promote the illegal break-repair– lead to a greater radiosensitivity of proliferating aged cells.

## Discussion

The study of low-dose responses in cell models relevant to human cancer is essential to a better understanding of the potential risks of medical irradiation procedures. Concerned about the risks of mammography screening in the adult population, we analyzed the ability of *in vitro* aged human mammary epithelial cells to cope with mammogram-induced DNA damage. Our study clearly shows that late passage mammary epithelial cells exposed to mammogram X-ray doses have a diminished capacity to cope with DNA damage, which is translated into an increased persistence of open DSBs, which finally promotes unfaithful repair and micronucleus formation.

The blind direct microscope enumeration of γH2AX foci in individual cells evidenced that X-ray doses equivalent to discrete mammogram explorations induce a significant increase of DSBs in mammary epithelial cells. Our data fits well with the sensitivity of the γH2AX foci assay, which is capable of detecting radiation doses down to a few mGy [7]. But, most importantly, we are reporting here that X-ray doses equivalent to a single mammogram exploration induced increased amounts of DSBs in the *in vitro* aged epithelial cells, but not in their young counterparts. Sedelnikova et al [17] did not observe any difference in γH2AX foci level with regard to donor age or cell-PD after a single acute dose of 600 mGy in peripheral lymphocyte and fibroblast samples. The observed difference between the two studies highlights the importance of the observation time-point election when caused damage is estimated from the analysis of protein foci having a marked dynamics of appearance and disappearance. We evaluated the mammography effects 2 h post irradiation, whereas Selenikova’s studies were performed only 30 minutes post-irradiation; this probably does not leave enough time for the age-related differences to show up. Moreover, uncertainty can arise when age-dependent carcinogenic risks are extrapolated from one cell type to another because, although DDR pathways are common to all higher eukaryotic cell types, they may not be equally efficient. Epithelial cells emerge from stress-induced senescence with dramatically higher probability than fibroblasts (10^−5^ vs 10^−9^) [26], thus potentially leading to higher risks of transformation. The study of the low-dose responses in epithelial cells is essential to a better understanding of potential carcinogenic risks. Our direct study using HMECs irradiated under a mammogram device revealed aging to be a key factor in the ability of cells to cope with mammogram-induced damage.

Comprehending the limitations of the DDR mechanisms and their impairment with cell aging is essential to optimize cancer-avoidance strategies. Here, we show that *in vitro* aged epithelial mammary cells are not as efficient as their young counterparts in recruiting 53BP1 after low-dose radiation exposure. Rather than a minimum level of DNA damage required to trigger an efficient response, the results of our time-course experiment of 53BP1 foci formation favor an explanation based on a delayed mobilization of DDR proteins in the late PD cell samples. An additional manifestation of the inefficient damage response of the *in vitro* aged epithelial cells is that a significant proportion of radiation-induced DSBs finally fails to recruit 53BP1. Altogether these results point to an inefficient damage response in the *in vitro* aged epithelial cells to low-dose radiation, which is due both to the delayed and incomplete mobilization of repair proteins to DSB sites. It is important to note that the age-related DDR efficacy decline observed in the present study occurs before the mammary epithelial cells enter senescent growth arrest. Therefore, this goes further than the well-defined deterioration in the homeostasis and functions of tissues associated with cellular senescence [27], [28], such as the delayed kinetics of DSB processing reported in senescing fibroblasts and peripheral blood lymphocytes [17]. Consistent with our results, Mao et al [29] recently showed that homologous recombination-mediated DSB repair declines sharply with increasing replicative age in proliferating fibroblasts. What causes *in vitro* aged cells to respond inefficiently to chromosome breaks? The most likely scenario is that chromatin or nuclear envelope changes could influence the mobilization of proteins involved in the DDR [30]. Age is associated with an overall increase in heterochromatin domains in murine and primate tissues [31] and with a deterioration of nuclear pore complexes [32]. All these age-associated changes might hinder mobilization of repair proteins to DNA lesions [33], [34] and compromise the exquisitely regulated DDR pathways.

In order to understand the transcendence of inefficient DSB processing, we have taken an additional step. The enumeration of γH2AX foci and the flow cytometric analysis of histone labeling at different times after low-dose X-ray exposure reveals that the inefficient damage response of *in vitro* aged mammary epithelial cells leads to delayed DSB disappearance. Retarded DSB repair is a direct consequence of cell aging rather than a consequence of the presence of dysfunctional telomeres, because, as shown here, telomerase reactivation does not rescue this deficient phenotype. Therefore, despite telomere erosion being an important factor for radiation sensitivity, cell aging *per se* also contributes to this. In late-passage epithelial mammary cells with slow response to DSB, many broken ends remain as repair substrates close in space and time. Although the repair pathways might subsequently process them, this delay would dramatically increase the probability of misrepair [24], [35]. Not surprisingly, an increase in the frequency of micronuclei is observed in the *in vitro* aged epithelial cells exposed to an X-ray dose equivalent to a single mammogram exploration (two view screens). Hence DSBs that have not been quickly detected by the DDR machinery and efficiently repaired will pose a higher risk for causing genomic rearrangements and chromosome instability. These results are consistent with recent IRCP published data that classifies breast tissue as amongst those that are most sensitive to radiation. Our findings of long-lasting and incomplete DSB repair in late passage human mammary epithelial cells constitutes a proof of their increased radiation sensitivity and might be related to increased carcinogenic risks of radiation exposures at older ages [9]. In the specific case of breast cancer, which has already been described as strongly dependent on hormonal and extracellular signals, age should be considered as an additional factor to be taken in account to properly evaluate the carcinogenic risks of radiation.

## Materials and Methods

### Cell Culture

Primary non-transformed human mammary epithelial cells (HMEC) derived from normal breast tissue of three Caucasian women age 28, 50 and 58; cells purchased from Gibco (donor 3), Cambrex Bio Science Walkersville (donor 1) and Cambrex Biowhittacker (donor 2), respectively. The cells were seeded into T 25 flask/chamberslides/plates depending on the experiment and were grown in serum-free MepiCGS (ScienCell, research laboratories) supplemented with penicillin and streptomycin at 37°C and 5% CO_2_. The number of accumulated population doublings (PD) achieved by the culture at each passage was determined using the equation PD = PD initial+log(N/N_0_), where N stands for the initial number of harvested cells, and N_0_ for the number of cells plated. Confluent cultures of early PD HMEC (PD 25–29 for donor 1, PD 27 for donor 2 and PD 24 for donor 3 HMECs) and late PD HMEC (PD 33–38, for donor 1, PD 38–40 for donor 2 and PD 32–34 for donor 3 HMECs) were used in experiments. A β-galactosidase assay was performed in order to score the fraction of senescent cells in each experiment. Less than 10% of early and late PD HMECs were senescent in all the experiments performed.

### Irradiation

HMEC cells were irradiated with one, two and ten automatic shots X-rays under a mammogram X-ray diagnostic device (SENO DMR plus, General electric). Each shot was equivalent to 10 mGy at skin surface (measured with an R-100 detector) and 2.7 mGy glandular dose. The X-ray molybdenum-anode tube voltage was 28 keV and a dose rate of 0.67 Gy/min was used. HMECs were exposed in a chamber slide support, placed on top of several methacrylate plates to emulate the average height of breast under compression. For γH2AX and 53BP1 proteins colocalization experiments, HMEC were irradiated at 1 Gy by exposing them to ^137^Cs γ-rays at a dose rate of 5.45 Gy/min using an IBL 437C source.

### Immunostaining

HMECs seed in chamber slides were grown until 70% confluence was reached. Fixation was carried out with paraformaldehid 4% for 10 minutes. Cells were then permeabilized in 1 × PBS-1% TritonX100 solution. The blocking step was carried out with 0.1% Tween20 and 2% fetal calf serum diluted in 1X PBS for 1 hour at room temperature. γH2AX and 53BP1 proteins were detected using mouse anti-γH2AX (Ser139) (Upstate) and rabbit anti-53BP1 (Abcam) at a final concentration of 1∶1000. Secondary antibodies were anti-mouse Cy3 (Amersham Biosciences) and anti-rabbit Alexa 568 (Molecular Probes). Three rounds of washes with PBS-0.1% Tween 20 were carried out to eliminate any excess of antibodies. Progressive alcohol dehydration was performed, followed by nuclear staining with 4′,6- diamidino-2-phenylindole (DAPI) for fluorescent counterstaining DNA for microscopy. DAPI was added at a final concentration of 2.5 µg/ml in Vectashield Mounting Medium. Fluorescence signals were visualized under an Olympus BX microscope equipped with epifluorescent optics specific for each fluorochrome. Images were captured and analysed using Cytovision software (Applied Imaging, Inc.).

### Flow Cytometric γH2AX Analysis

Detection of *γ*H2AX signal was carried out by using the γH2AX phosphorylation assay kit for flow cytometry (Upstate Biotechnology, Lake Placid, NY). The assay was performed following manufacturer instructions with two major modifications: the FITC-labeled antibody was incubated overnight at 4°C and an extra 5 min permeabilitzation step with 0.5% TritonX100 was also included. Cells were suspended in flow buffer (1%f PBS 1% RNaseA containing 20 ml PI) and analyzed by using a FACS Caliber Flow Cytometer (Becton Dickinson). Data were analyzed by using both Cell Quest and Cyflogic software (Tree Star, San Carlos, CA). In order to increase the sensitivity of the flow cytometric detection of γH2AX positive cells, the mean number of cells positive for γH2AX in G1, S and G2/M-phase in untreated cells was subtracted from the respective means of the G1, S and G2/M subpopulations of the radiation-exposed cells. We also used the isotype control to estimate the non-specific antibody-binding component, since this component varies for the untreated and treated cells. It is noteworthy that TUNEL positive cells were below 5% in all samples, thus excluding the possibility that the increased amount of γH2AX labeling was caused by apoptosis.

### Micronucleus Assay

Irradiated and non-irradiated cells grown in chamberslides were cultured for 24 h in the presence of cytochalasin B at a final concentration of 3 µg/ml. The cells were washed twice in 1% PBS for 1 minute, after which they were placed in 0.075 mM KCl hypotonic solution at 37°C and fixed in acetic acid/methanol for 15 min. After removing the fixative, the slides were completely air dried and stained with DAPI (0,25 µg/ml) and PI (40 µg/ml) in order to identify the binucleated cells.

### PNA-FISH of Metaphase Chromosomes

Chromosome metaphase preparations were obtained through a colcemid treatment (0.02 µg/ml final concentration) for 8h before harvesting, followed by hypotonic shock (0,075M KCL, 37°C for 30 min) and subsequently fixed with a mixture of glacial acetic acid and methanol (25%:75%), dropped onto the ice-cold slides and air-dried. Slides were stored at −4°C before being labeled with PNA-FISH probes using a a Cy3-(CCCTAA)3 PNA-probe for telomeres and a FITC-AAACACTCTTTTTGTAGA PNA-probe for centromeres (PE Biosystems; Foster City, CA), as previously described [36]. To evaluate telomere dysfunction DAPI was added (2.5 µg/ml final concentration) and chromosome counting was performed in all the metaphases before FISH-PNA labeling. Subsequently, pantelomeric probes allowed us to determine the chromosome arms that had telomere-signal-free ends (TSFE). The TSFE rate was calculated by dividing the number of chromosome arms without a telomere signal by the number of scored metaphases for each cell population analyzed. Images were captured and analysed using Cytovision software (Applied Imaging, Inc.).

### Transduction Procedures

To reconstitute telomere length, late PD HMECs from donor 1 were transduced with viral particles containing LV.hTERT, a lentivirus construct provided by the Viral Vector Facility (CNIC, Spain), in the presence of 4 µg/ml Polybrene (Sigma-Aldrich). After 24 h post-transduction, medium was replaced and cells were incubated at 37°C and 5% of CO_2_ atmosphere. To evaluate telomerase activity, protein extracts were prepared from transduced and control cells using a RIPA lysis buffer. Protein concentration was measured with a spectrophotometer (NanoDrop 2000). The presence of hTERT, necessary for telomerase activity, was confirmed by Western blot immunodetection.
